# Glucocorticoids contribute to metabolic and liver impairments induced by lactation overnutrition in male adult rats

**DOI:** 10.3389/fphys.2023.1161582

**Published:** 2023-05-10

**Authors:** Camila F. de Souza, Larissa Rugila S. Stopa, Andressa B. Martins, Ana Luiza M. Wunderlich, Gabriela Mendicelli Lopes, Flaviane de Fatima Silva, Ayumi Cristina Medeiros Komino, Dimas A. M. Zaia, Cassia Thaïs B. V. Zaia, Fabio Bessa Lima, Ernane Torres Uchoa

**Affiliations:** ^1^ Multicenter Postgraduate Program in Physiological Sciences, State University of Londrina, Londrina, Brazil; ^2^ Postgraduate Program in Physiological Sciences, State University of Londrina, Londrina, Brazil; ^3^ Department of Physiological Sciences, State University of Londrina, Londrina, Brazil; ^4^ Department of Physiology and Biophysics, University of Sao Paulo, Sao Paulo, Brazil; ^5^ Department of Chemistry, State University of Londrina, Londrina, Brazil

**Keywords:** adrenalectomy, corticosterone, litter size reduction, triglycerides, obesity

## Abstract

**Introduction:** Lactation overnutrition is a programming agent of energy metabolism, and litter size reduction leads to the early development of obesity, which persists until adulthood. Liver metabolism is disrupted by obesity, and increased levels of circulating glucocorticoids are pointed as a possible mediator for the obesity development, since bilateral adrenalectomy (ADX) can reduce obesity in different models of obesity.

**Methods:** This study aimed to evaluate the effects of glucocorticoids on metabolic changes and liver lipogenesis and insulin pathway induced by lactation overnutrition. For this, on the postnatal day 3 (PND), 3 pups (small litter—SL) or 10 pups (normal litter—NL) were kept with each dam. On PND 60, male Wistar rats underwent bilateral adrenalectomy (ADX) or fictitious surgery (sham), and half of ADX animals received corticosterone (CORT- 25 mg/L) diluted in the drinking fluid. On PND 74, the animals were euthanized by decapitation for trunk blood collection, and liver dissection and storage.

**Results and Discussion:** SL rats presented increased corticosterone, free fatty acids, total and LDL-cholesterol plasma levels, without changes in triglycerides (TG) and HDL-cholesterol. The SL group also showed increased content of liver TG, and expression of fatty acid synthase (FASN), but decreased expression of PI3K_p110_ in the liver, compared to NL rats. In the SL group, the ADX decreased plasma levels of corticosterone, FFA, TG and HDL cholesterol, liver TG, and liver expression of FASN, and IRS2, compared to sham animals. In SL animals, CORT treatment increased plasma levels of TG and HDL cholesterol, liver TG, and expression of FASN, IRS1, and IRS2, compared with the ADX group. In summary, the ADX attenuated plasma and liver changes observed after lactation overnutrition, and CORT treatment could reverse most ADX-induced effects. Thus, increased circulating glucocorticoids are likely to play a pivotal role in liver and plasma impairments induced by lactation overnutrition in male rats.

## Introduction

The nutritional or hormonal setting during fetal and/or postnatal periods plays an important modulatory role. It is known that the development of several organs is not completed at birth and it often continues during the lactation period (Ellsworth et al., 2018). So, this window may be considered a vulnerable time for offspring, once the systems are plastic and sensitive to changes in the environment, which will result in effects on adult life, as well-established by the “Developmental origins of health and disease” (DOHaD) concept (Barker, 1998).

Food intake during the neonatal period is a relevant factor for the development of obesity in adulthood (Fiorotto et al., 1991). In this context, the litter size reduction method in rodents is an important tool to observe the long-term effects of lactation overnutrition as demonstrated for the first time by Kennedy, 1957 (Plagemann, 2006). This method consists of allocating the pups in smaller litters (SL), with 3 pups/dam compared with the control with 10 pups/dam. Then, the animals from SL develop earlier obesity which persists until adulthood (Plagemann, 2006; Spencer, 2013).

One of the comorbidities of obesity is triglycerides (TG) accumulation in the liver, which occurs from an imbalance between lipid input, as fatty acid uptake and *de novo* lipogenesis (DNL), and lipid output as very low-density lipoprotein (VLDL) (Donnelly et al., 2005; Bradburry, 2006; Doege and Stahl, 2006). Under physiological conditions, the concentration of hepatic TG is low, since the liver is not a fat storage organ. Therefore, in a condition of excess of carbohydrates, the liver produces fatty acids by DNL and exports triglyceride as VLDL (Donnelly et al., 2005; Doege and Stahl, 2006; Bradburry, 2006; Nestel et al., 1962; Cohen et al., 2011; Kawano and Cohen, 2013).

Obesity is associated TG accumulation in the liver, once the lipid storage capacity of an expanded adipose tissue is limited, and the excess of lipids, and TG are stored in hepatocytes. The sources of TG in the liver are increased lipolysis (approximately 60%) and DNL in the liver (25%) (Donnelly et al., 2005). In this context, the DNL consists in synthesizing non-esterified fatty acids from glucose. For this, glucose is converted to acetyl-CoA through glycolysis, and acetyl-CoA is then converted to malonyl-CoA by acetyl-CoA carboxylase (ACC). Fatty acid synthase enzyme (FASN) catalyzes the formation of palmitic acid from malonyl-CoA and acetyl-CoA. Palmitic acid is then elongated and desaturated to generate monounsaturated fatty acids, which are the major fatty acid constituents of TG. Acyl-CoAs are esterified to form diacylglycerol, which is esterified to another acyl-CoA molecule to form triglyceride by the acyl-CoA diacylglycerol acyltransferase (DGAT), which seems to be especially important for VLDL production (Wurie et al., 2012; Heeren and Scheja, 2021). Another important molecule in DNL is the Apolipoprotein B100 (Apo B100), which is synthesized by hepatocytes and has a unique feature of the ApoB molecule the ability to interact with lipid species (phospholipids, cholesterol, TG, and cholesteryl esters) used to arrangement of VLDL (Rutledge et al., 2010).

Lipid accumulation in the liver has a relationship with fetal and neonatal programming impairment in liver lipid metabolism (Oben et al., 2010). Animals with childhood obesity present metabolic liver dysfunctions such as TG accumulation (Oben et al., 2010), increased circulating free fatty acids, hyperinsulinemia, impairment of the insulin signaling pathway in the liver and skeletal muscle, in addition to a higher concentration of circulating glucocorticoids (Hou et al., 2011), once that hypothalamic-pituitary-adrenal axis (HPA) of these animals matured more quickly (Boullu-ciocca et al., 2005; Spencer and Tilbrook, 2009). According to the literature, adrenal glucocorticoids have an important role in energy homeostasis. Glucocorticoids are known to increase appetite and body weight in humans and rodents (Tataranni et al., 1996; Zakrzewska et al., 1999), and their excess can increase central adiposity, as seen in Cushing’s syndrome (Rebuffé-Scrive et al., 1988). Different models of obesity have increased plasma concentrations of glucocorticoids, and conversely, bilateral removal of the adrenal glands, adrenalectomy (ADX), can attenuate or prevent the development of obesity (Bruce et al., 1982; Yukimura et al., 1978; Dubuc and Wilden, 1986). Within lipid metabolism, glucocorticoids act in the synthesis and oxidation of fatty acids, the esterification of fatty acids to triglyceride and phospholipid, and the outward hepatic transport of lipoprotein lipids (Klausner and Heimberg, 1957).

Thus, it is known that: 1) glucocorticoids are pointed as possible mediators for obesity development and its comorbidities in different experimental models; 2) animals with obesity due to litter reduction show metabolic liver dysfunctions and increased circulating glucocorticoids; 3) the litter size reduction method is a model that leads to obesity and its comorbidities; 4) ADX reverses several anabolic effects in models of obesity. Therefore, this study hypothesized that glucocorticoids contribute to dyslipidemia and metabolic liver dysfunctions in adult male rats induced by neonatal obesity. For the investigation of this hypothesis, the current work aimed to evaluate the effects of ADX on biometric parameters, plasma lipid profile, lipid metabolism and insulin signaling pathway in the liver in adult male animals reared in small litters.

## Materials and methods

### Animals

Male Wistar rats (n = 77) were obtained from the mating of 37 females with males from the Animal Facility of State University of Londrina (UEL). Of these 37 females, the pregnancy was confirmed by the presence of sperm on the vaginal smear of 33 females (89%). The adjustment of the litter size occurred on the postnatal day (PND) 3, with PND 0 considered the day of birth. The lactation overnutrition was induced remaining with dam 3 pups, 2 males and 1 female to compose the small litter (SL), and the normal litter (NL) remained with dam 10 pups, 5 males and 5 females (Stopa et al., 2021). The surplus pups were anesthetized with an association of ketamine (100 mg/kg, 10%, Agener União, Apucarana, Brazil) and xylazine hydrochloride (20 mg/kg, 2%, Anasedan^®^, Vetbrands, Jacareí, Brazil) intraperitoneally, and euthanized by decapitation. After weaning, the dams were also anesthetized with the above-mentioned association of ketamine and xylazine hydrochloride intraperitoneally, and euthanized by decapitation, the female pups were used in other experiments and the male pups were used in the current study and were housed in groups of 3–4 rats of the same experimental group in each cage. The animals were kept in controlled conditions of light (12 h light/dark cycle: 6 a.m. to 6p.m./6 p.m. to 6 a.m.) and temperature (22 ± 2°C), with fluid and feed *ad libitum*, except for the hours of feed restriction before the euthanasia. All experiments were performed at the Department of Physiological Sciences/UEL. The experimental procedures were approved by the Ethics Committee on Animal Use for experimentation (CEUA number: 3457.2109.11, Of. Circ. CEUA 60/2019).

### Adrenalectomy or fictitious surgery (sham)

Bilateral ADX and sham surgeries were performed under anesthesia with the association of ketamine (100 mg/kg, Agener União) and xylazine hydrochloride (20 mg/kg, Anasedan^®^, Vetbrands, Jacareí, Brazil, 2%) intraperitoneal, and a single dorsal midline incision on the skin and a bilateral small cut through the muscle layer was made. After the surgery and during the experimental period, ADX animals were given 0.9% saline with 0.5% ethanol, without corticosterone (ADX) or with corticosterone (B: corticosterone, Sigma Co., CA) (ADX + B) at the concentration of 25 mg/L (Uchoa et al., 2009a; Uchoa et al., 2009b; Uchoa et al., 2010; Uchoa et al., 2012). There was about 15% of mortality of animals submitted to ADX surgery during or after the first days of the procedure. Sham-operated animals underwent similar surgical procedures without removal of adrenal glands and were given tap water with 0.5% ethanol to drink.

### Experiment protocol

**FIGURE 1:**
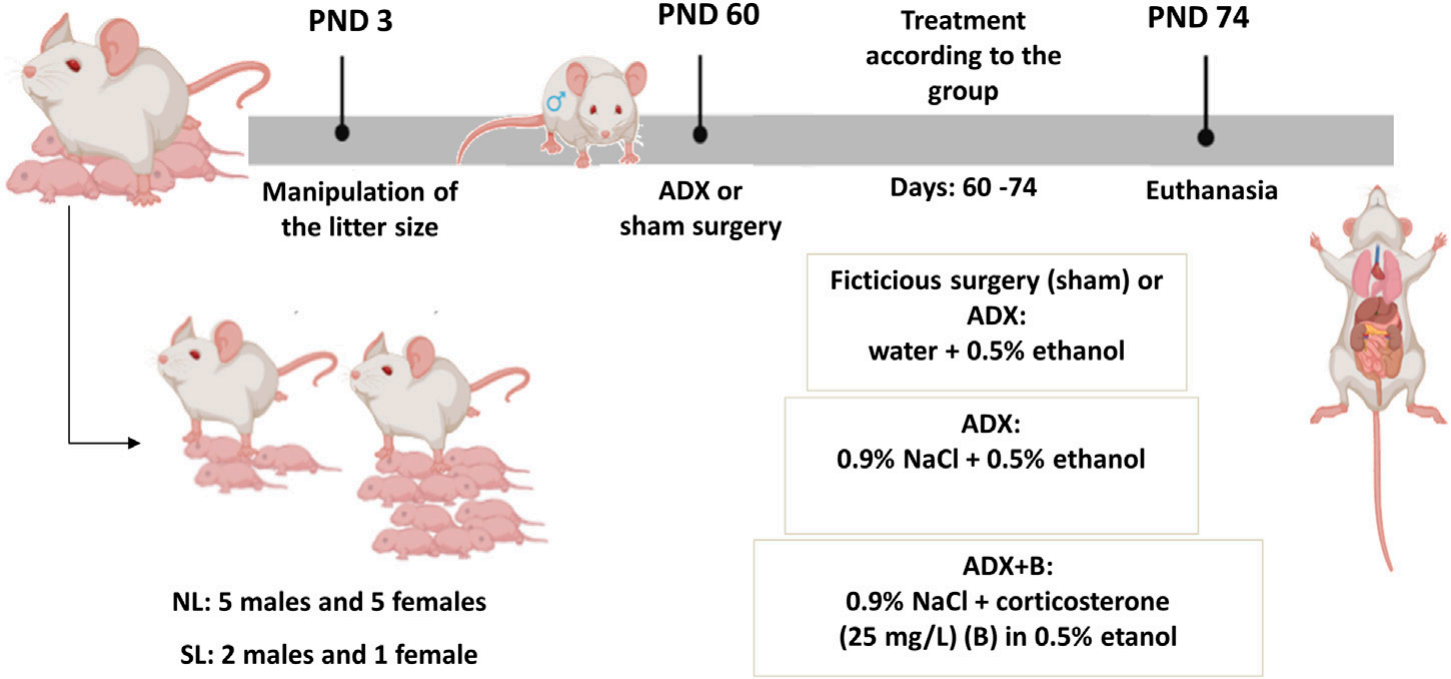
Experimental design of protocol. ADX: adrenalectomy; NL: normal litter; SL: small litter; PND: postnatal day.

On PND 60, animals of both litters were submitted to bilateral adrenalectomy (ADX) or sham surgery (fictitious surgery). After the surgery, the animals were kept with access to the solution of water containing 0.5% ethanol, or 0.9% NaCl containing 0.5% ethanol, or 0.9% NaCl containing corticosterone (25 mg/L) diluted in ethanol 0.5% for 14 days, according to the experimental group (NL-*sham*; SL-*sham;* NL- ADX; SL-ADX; NL- ADX + B and SL-ADX + B). All animals were weighed, and the amount of food ingested was daily evaluated for a period of 14 days. On PND 74, the rats were weighed, and the nasoanal length was measured, for Lee index determination (Bernardis and Patterson, 1968), and the animals were kept under food restriction from 8 a.m. to 2 p.m. and non-anesthetized animals were euthanized by decapitation at 2 p.m. (Figure 1). After euthanasia, blood was collected to assess plasma concentrations of corticosterone, free fatty acids (FFA), TG, total, HDL and LDL cholesterol. The liver was removed, and the left lobe was stored for TG content and western blotting analyses.

### Euthanasia

For the purpose to avoid hormonal and biochemical changes induced by anesthesia (Guarino et al., 2013; Windeløv et al., 2016), the euthanasia was performed by decapitation in non-anesthetized animals after 6 h feed restriction, as approved by the Ethics Committee on Animal Use for experimentation (CEUA number: 3457.2109.11, Of. Circ. CEUA 60/2019). The blood was immediately collected in heparinized tubes and centrifuged at 14,000 ×*g* for 20 min at 4°C to plasma obtention, which was stored at −20°C for biochemical analysis.

### Measurement of plasma concentration of corticosterone, FFA, TG, total, HDL, and LDL cholesterol

The determination of plasma concentrations of total cholesterol, HDL cholesterol (commercial kits, VIDA biotechnology, MG), LDL cholesterol (commercial kits, Wiener lab., Argentina), TG (commercial kits, Laborclin, PR) and FFA (Falholt et al., 1973) were performed by the spectrophotometric method. The Fluorimetric method of Guillemin et al. (1958) was used to determine corticosterone (de Souza et al., 2019; Stopa et al., 2019; Stopa et al., 2021).

### Analysis of liver TG content

To measure the liver TG content, lipids were extracted from liver samples with chloroform-methanol by the Folch method (Folch et al., 1957) as previously described (de Fatima Silva et al., 2022). The TG were determined by enzymatic assay (commercial kit, Labtest, Lagoa Santa, MG, Brazil) and expressed by mg of TG by 100 mg of the liver.

### Protein expression by western blotting in the liver

Liver samples were homogenized in extraction buffer (135 mM NaCl, 2.7 mM KCl, 1 mM MgCl_2_, 10 mM EDTA, 5 mM Na_4_P_2_O_7_, 10 mM NaF, 1% Triton X-100, 10% Glycerol, 20 mM Tris, 2 mM PMSF, 2.5 mM Na_3_VO_4_, cOmplete™ tablets, and pH 7.4) to obtain the total protein fraction. The homogenate was centrifuged (12.000 rpm, 40 min, 0°C), and the total proteins of the supernatant were quantified by the Bradford method (Bradford, 1976).

In all analyzes, equal amounts of total proteins (35 µg) plus Laemmli buffer (BioRad, #1610747) were added to 8% polyacrylamide gel, submitted to electrophoresis, and transferred to nitrocellulose membrane. The membranes were blocked with 4% BSA diluted in phosphate-buffered saline with 0.1% Tween 20 (PBST) and incubated overnight (4°C) with the primary antibody diluted in 4% BSA in PBST, as detailed in Supplementary File S2. On the next day, membranes were rinsed (4 × 5 min) with PBST, followed by 75 min incubation with secondary antibody conjugated to peroxidase (rabbit IgG, Jackson ImmunoResearch, #111-035-003; mouse IgG, Jackson ImmunoResearch, #115-035-003; or goat IgG, Jackson ImmunoResearch, #305-035-003; all secondaries 1:5000 diluted in 0.1% PBST; room temperature). A new 4 × 5 min 0.1% PBST rinse protocol was performed and the membranes were incubated for 2 min with peroxidase substrate (Clarity Western ECL, BioRad, #1705061) for chemiluminescence detection (G:BOX, Syngene™). The density of the blots was analyzed in ImageJ software (National Institutes of Health, United States of America) and expressed in arbitrary units after normalization by the constitutive protein vinculin. Different targets were obtained from different membranes, except for the phosphorylated proteins, where after the incubation with the anti-phospho antibodies, the respective membrane was stripped, blocked, and incubated with the total antibody for each protein. For each membrane the control blot vinculin was performed, to assure the sample loading and provide accurate normalization (Supplementary File S1).

### Statistical analysis

The normal distribution and homogeneity of the data were tested by Shapiro-Wilk test and Levene’s test, respectively, and the results were analyzed by two-way ANOVA, followed by the Tukey *post hoc* test. Data are expressed as mean ± standard error of the mean (SEM). Differences were considered significant at *p* < 0.05.

## Results

**FIGURE 2:**
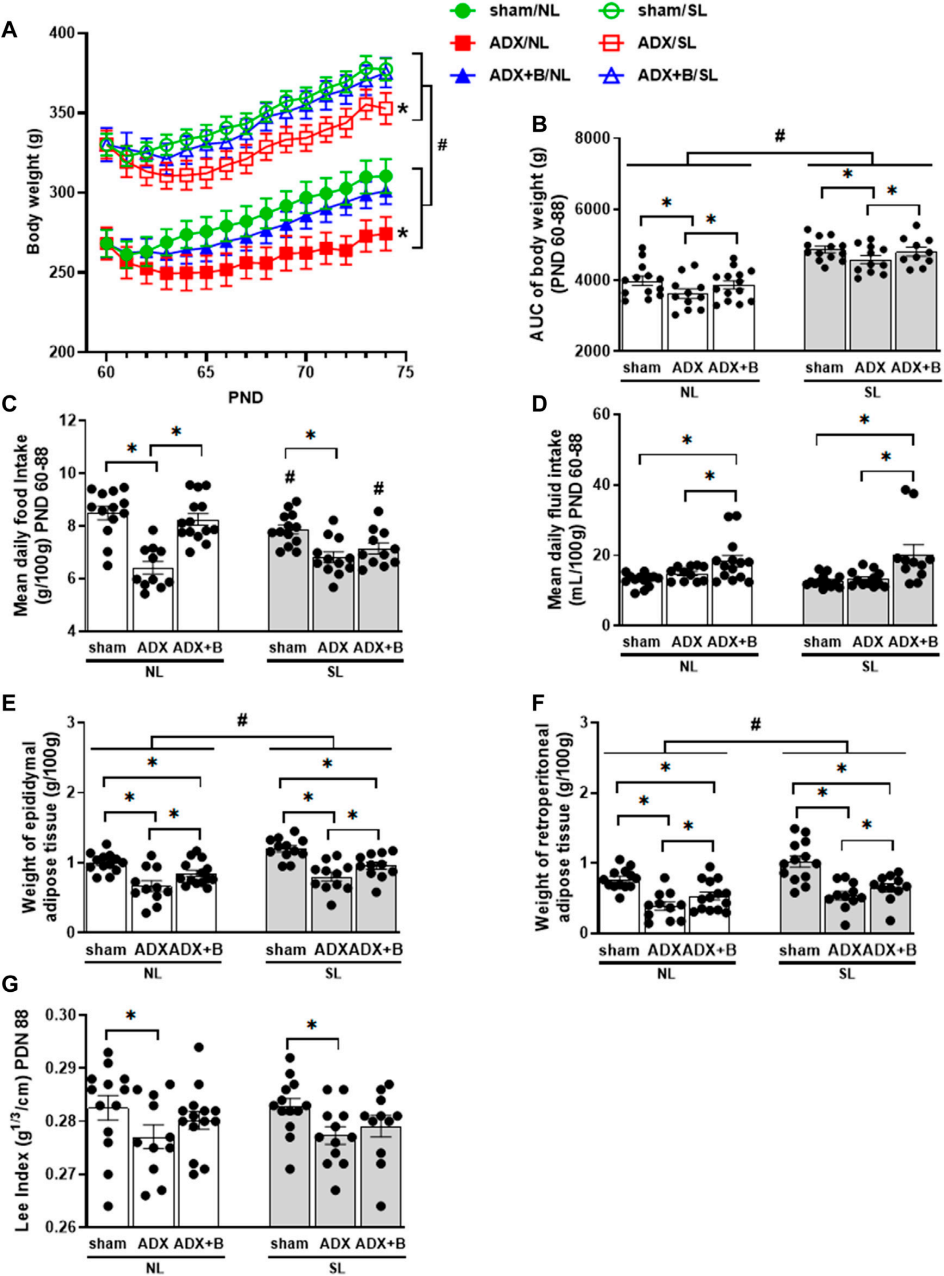
Body weight from PND 60 to 74 **(A)**, AUC of body weight from PND 60 to 74 **(B)**, mean daily food intake **(C)** and mean daily fluid intake (mL/100 g) from PND 60 to 74 **(D)**, weights of epididymal **(E)** and retroperitoneal **(F)** adipose tissues on PND 74, and Lee Index (g ⅓/cm) on PND 74 **(G)** of male rats from normal (NL) and small (SL) litters: sham; ADX; ADX + B. Data were analyzed by two-way Anova, followed by the Tukey *post hoc* test and expressed as mean ± SEM. *p < 0.05; #*p* < 0.05 SL *versus* NL; B = corticosterone (n = 13).

Adrenalectomy attenuates changes in ponderal, and biochemical parameters induced by lactation overnutrition in male rats:

The body weight along the experimental protocol showed no interaction among days (PND 60–74), litter size (NL and SL) and groups (sham, ADX, and ADX + B) [F (28;924) = 0.888, *p* = 0.532], but with effects of days [F (14;53) = 63.395, *p* < 0.0001], litter size [F (1;66) = 89.42, *p* < 0.0001] and groups [F (2;66) = 3.641, *p* = 0.032]. This body weight response was integrated into the area under the curve (AUC) of body weight, which showed no interaction between the groups and litter size on the AUC of body weight [F (2;66) = 0.055, *p* = 0.946], with effects of litter size [F (1;66) = 89.6, *p* < 0.0001] and groups [F (2;66) = 3.83, *p* = 0.027]. SL animals demonstrated an increase in body weight compared with NL groups. ADX animals reduced body weight compared with the sham group and the treatment with corticosterone increased body weight, compared with the ADX group in both litters (Figures 2A,B).

An interaction was observed between groups and litter size on food intake [F (2;68) = 5.88, *p* = 0.004]. Food intake of SL animals of sham and ADX + B groups was lower than shown in NL groups. ADX groups in both litters showed a reduction in food intake, compared with sham animals, and treatment with corticosterone reversed ADX effects in NL animals (Figure 2C). No interaction was observed between groups and litter size on fluid intake [F (2;68) = 1.31, *p* = 0.28], without effects of litter size [F (1;68) = 2.05, *p* = 0.16] and with effects of groups [F (2;68) = 15.33, *p* < 0.001]. ADX + B groups in both litters showed increased fluid intake compared with ADX and sham animals (Figure 2D).

No interaction was observed between the groups and litter size on the weights of epididymal [F (2;66) = 0.394, *p* = 0.676] and retroperitoneal [F (2;67) = 0.668, *p* = 0.516] adipose tissues, with effects of litter size for epididymal [F (1;66) = 11.46, *p* < 0.001] and retroperitoneal depots [F (1;67) = 12.2, *p* < 0.001], as well as with effects of groups for epididymal [F (2;66) = 23.6, *p* < 0.0001] and retroperitoneal depots [F (2;67) = 27.3, *p* < 0.0001]. SL animals showed increased weights of both fat depots compared with NL groups. ADX animals reduced the weights of epididymal and retroperitoneal adipose tissues compared with the sham group and the treatment with corticosterone increased the weight of these fat depots, compared with the ADX group in both litters (Figures 2E,F). No interaction was observed between the groups and litter size on Lee index [F (2;68) = 0.09, *p* = 0.914], with no effects of litter size [F (1;68) = 0.015, *p* = 0.903] and with effects of groups [F (2;68) = 4.11, *p* = 0.021]. ADX groups in both litters demonstrated a decrease in the Lee index compared with their respective sham animals (Figure 2G).

**FIGURE 3:**
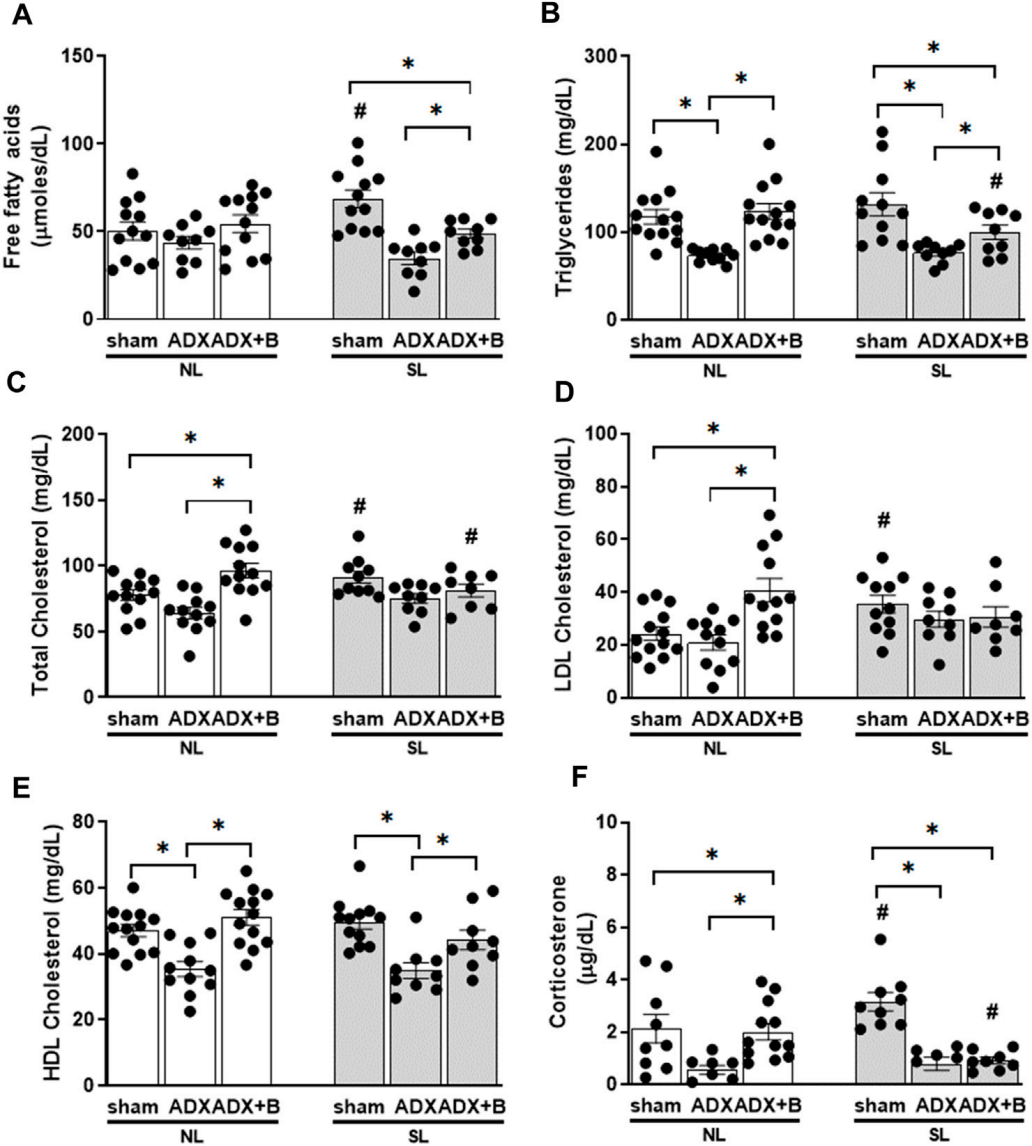
Plasma concentrations of free fatty acids **(A)**, triglycerides **(B)**, total cholesterol **(C)**, HDL cholesterol **(D)**, LDL cholesterol **(E)** and corticosterone **(F)** of male rats from normal (NL) and small (SL) litters on PND 74: sham; ADX; ADX + B, from PND 60 to 74. Data were analyzed by two-way Anova, followed by the Tukey *post hoc* test and expressed as mean ± SEM. *p < 0.05; #*p* < 0.05 SL *versus* NL; B = corticosterone (n = 13).

There was an interaction between groups and litter size on FFA [F (2;57) = 5.59, *p* = 0.006] and TG [F (2;59) = 3.29, *p* = 0.044] plasma levels. In sham animals, SL induced an increase in FFA plasma levels, and ADX reduced this response, without the effects of corticosterone treatment. ADX reduced plasma levels of TG and corticosterone treatment reversed this response in both litters, but TG levels were lower in ADX + B animals of SL than ADX-SL and ADX + B-NL groups. (Figures 3A,B).

An interaction was observed between groups and litter size (NL and SL) on plasma levels of total [F (2;56) = 5.54, *p* = 0.006] and LDL cholesterol [F (2;58) = 5.52, *p* = 0.006], without interaction in HDL cholesterol [F (1;61) = 2.22, *p* = 0.117]. The reduction the litter size promoted higher plasma levels of total and LDL cholesterol in the sham group compared to NL-sham animals. In the NL group, the treatment with corticosterone in ADX group induced higher concentrations of total and LDL cholesterol than in sham and ADX group (Figures 3C,D). Plasma levels of HDL cholesterol showed effects of the group [F (2;61) = 19.72, *p* < 0.001], without effects of the litter size [F (1;61) = 0.7, *p* = 0.407]. ADX reduced (*p* < 0.001) HDL plasma levels, compared to the sham group, and corticosterone treatment reversed (*p* < 0.001) this effect in both litters (Figure 3E).

Interaction was observed between group and litter size on the plasma concentrations of corticosterone [F (2;47) = 5.16, *p* = 0.009)]. Reduction of the litter size induced higher (*p* < 0.05) plasma concentrations of corticosterone in sham animals compared to their respective NL animals. Adrenalectomy reduced (*p* < 0.05) plasma concentration of corticosterone in both litters and treatment with corticosterone enhanced the plasma concentration of corticosterone only in NL animals, without effects in SL animals (Figure 3F).

**FIGURE 4:**
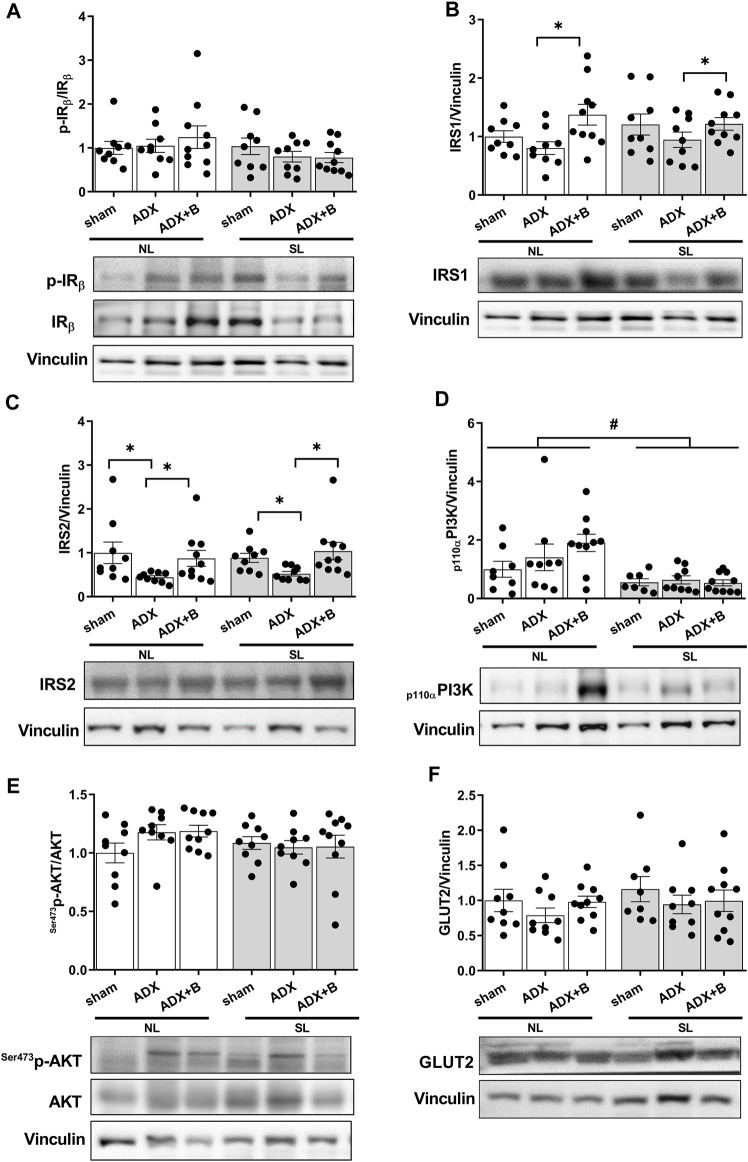
Liver expression of insulin receptor (IR) **(A)**, insulin receptor substrate (IRS) 1**(B)**, insulin receptor substrate (IRS) 2 **(C)**, PI3Kp110 **(D)**, Ser473pAKT/AKT **(E)** and GLUT2 **(F)** of male rats from normal (NL) and small (SL) litters on PND 74: sham; ADX; ADX + B. Data are expressed as mean ± SEM. *p < 0.05; #*p* < 0.05 SL *versus* NL; B = corticosterone (n = 13).

Effects of adrenalectomy and treatment with glucocorticoid on liver insulin signaling pathway of adult male rats with lactation overnutrition:

No interaction was observed between the groups and litter size on pIR/IR [F (2;50) = 1.08, *p* = 0.35], with no effects of litter size [F (1;50) = 2.56, *p* = 0.12] and groups [F (2; 50) = 0.18, *p* = 0.84] (Figure 4A). There was also no interaction between the groups and litter size on IRS1 [F (2;50) = 1.00, *p* = 0.37], with no effects of litter size [F (1;50) = 0.32, *p* = 0.57] and with effects of groups [F (2;50) = 4.67, *p* = 0.014] (Figure 4B). Treatment with corticosterone in ADX animals promoted higher expression of IRS1 in the liver of both litters. Interaction was not observed between the groups and litter size (NL and SL) on IRS2 [F (2;50) = 0.13, *p* = 0.88], with no effects of litter size [F (1;50) = 2.55, *p* = 0.12] and with effects of groups [F (2;50) = 12.59, *p* < 0.001]. ADX animals of both litters showed a decrease in expression of IRS2, compared to sham, and treatment with corticosterone reversed this response (Figure 4C).

Interaction was not observed between the groups and litter size on ^Ser473^pAKT/AKT [F (2; 50) = 1.41, *p* = 0.25], with no effects of litter size [F (1;50) = 1.71, *p* = 0.20] and groups [F (2; 50) = 1.21, *p* = 0.31] (Figure 4D). There was no interaction between the groups and litter size on _p110α_PI3K [F (2;47) = 1.72, *p* = 0.19], with effects of litter size [F (1;47) = 15.76, *p* < 0.001] and with no effects of groups [F (2;47) = 0.88, *p* = 0.42] (Figure 4E). The reduction of the litter size promoted a decrease in the expression of _p110α_PI3K compared with NL animals. No interaction was also between the groups and litter size on GLUT 2 [F (2;49) = 0.19, *p* = 0.83], without effects of litter size [F (1;49) = 0.99, *p* = 0.33] and groups [F (2;49) = 1.19, *p* = 0.31] (Figure 4F).

**FIGURE 5:**
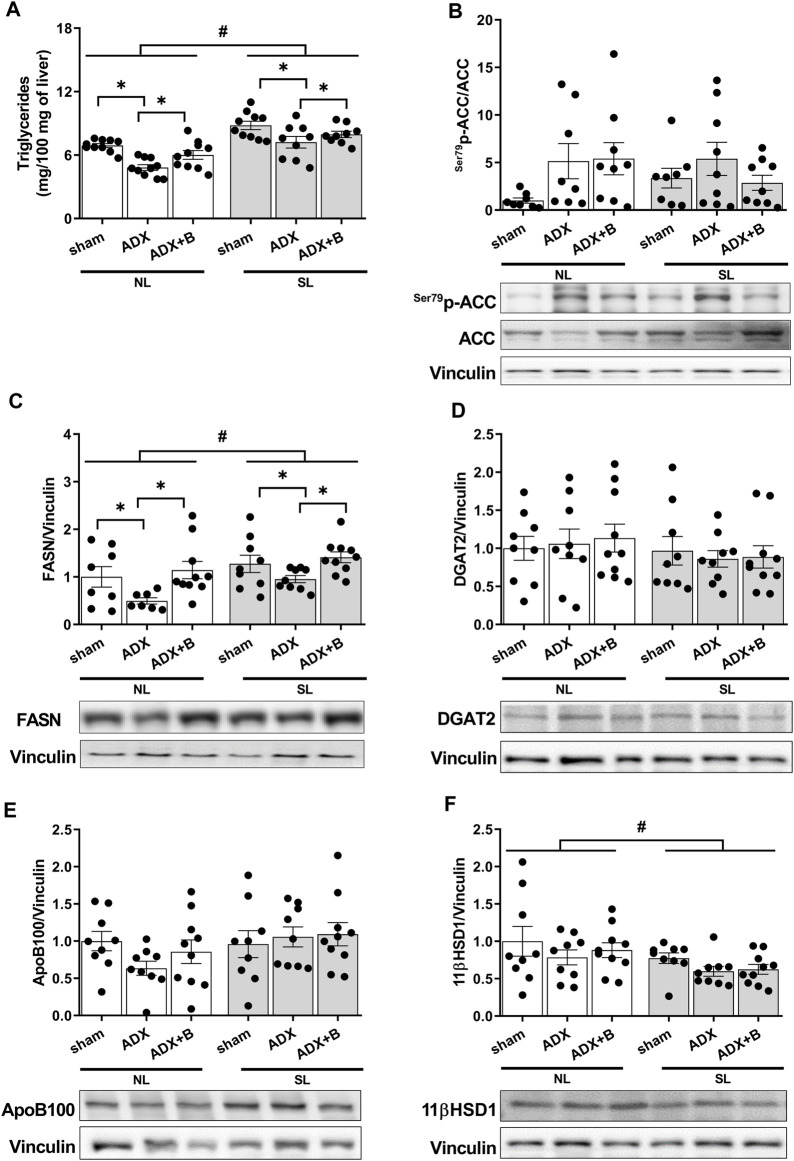
Liver triglycerides (mg/100 mg of liver) **(A)**, liver expression of fatty acid synthase (FASN) **(B)**, acetyl-CoA carboxylase (p-ACC/ACC) **(C)**, diacylglycerol acyltransferase (DGAT) 2 **(D)**, Apo B100 **(E)** and 11 β- Hydroxysteroid dehydrogenase 1 (11 βHSD1) **(F)** of male rats from normal (NL) and small (SL) litters on PND 74: sham; ADX; ADX + B. Data are expressed as mean ± SEM. *p < 0.05; #*p* < 0.05 SL *versus* NL; B = corticosterone (n = 13).

Effects of adrenalectomy and treatment with glucocorticoid on lipid metabolism in the liver of adult male rats with lactation overnutrition:

No interaction was observed between the groups and litter size on liver TG [F (2;52) = 0.27, *p* = 0.76], with effects of litter size [F (1;52) = 48.73, *p* < 0.001] and groups [F (2;52) = 12.73, *p* < 0.001]. SL groups presented higher (*p* < 0.05) liver TG content than NL animals. ADX promoted reduction (*p* < 0.05) in liver TG content and the treatment with corticosterone reversed this response in both litters (Figure 5A).

There was no interaction between the groups and litter size on p-ACC/ACC [F (2;45) = 1.60, *p* = 0.21], with no effects of litter size [F (1;45) = 0.0003, *p* = 0.99] and groups [F (2;45) = 2.49, *p* = 0.21] (Figure 5B). No interaction was observed between the groups and litter size (NL and SL) on FASN [F (2;47) = 0.24, *p* = 0.79], with effects of litter size [F (1;47) = 7.01, *p* = 0.011] and groups [F (2;47) = 6.82, *p* = 0.003] (Figure 5C). The reduction of the litter size promoted an increase of FASN expression, compared with NL animals, while the adrenalectomy reduced the FASN expression, and the treatment with corticosterone reversed this parameter in both litters.

Additionally, there was no interaction between the groups and litter size (NL and SL) on DGAT2 [F (2;50) = 0.06, *p* = 0.94], with no effects of litter size [F (1;50) = 1.92, *p* = 0.17] and groups [F (2;50) = 0.06, *p* = 0.94] (Figure 5D). Also, no interaction was observed between the groups and litter size (NL and SL) on APOB100 [F (2;50) = 1.22, *p* = 0.30], with no effects of litter size [F (1;50) = 2.96, *p* = 0.092] and groups [F (2;50) = 0.53, *p* = 0.59] (Figure 5E).

Finally, no interaction was observed between the groups and litter size (NL and SL) on 11β hydroxysteroid dehydrogenase 1 (11βHSD1) [F (2; 50) = 0.05, *p* = 0.95], with effects of litter size [F (1; 50) = 6.27, *p* = 0.02] and without effects of groups [F (2;50) = 1.64, *p* = 0.20] (Fig. 6 A). All SL animals showed decreased 11βHSD1 expression compared with NL groups (Figure 5F).

## Discussion

The present study aimed to investigate the glucocorticoids’ contribution to metabolic and lipidic liver functions in adult male Wistar rats with lactation overnutrition. For this purpose, the effects of ADX and the treatment with corticosterone were analyzed as tools to reach these goals. Indeed, this study is the pioneer to show that ADX attenuated plasma and liver changes observed after lactation overnutrition, and corticosterone treatment reversed most of the ADX-induced effects, indicating that circulating glucocorticoids contribute to liver and plasma impairments induced by childhood obesity in adult male rats.

There are different models to achieve obesity in rodents, and one of them is to observe the consequences of early neonatal overfeeding using the litter size reduction method (Plagemann et al., 1999a; Plagemann et al., 1999b). In this model, there is a decrease in competition for milk during the breastfeeding period and thus, several authors also affirm that the composition of breast milk is modified, presenting a higher concentration of lipids, mainly an increase in TG (Cunha et al., 2009; Moreira et al., 2009; Šefčíková et al., 2011).

The litter size reduction method programs overweight in adulthood according to several data (Plagemann et al., 1992; Boullu-Ciocca et al., 2005; Rodrigues et al., 2009; Conceição et al., 2011; Ferretti et al., 2011; Rodrigues et al., 2011). The increased body weight, adiposity and the impairment of lipidic profile in SL animals corroborates with the literature, where animals reared in small litters present obesity-related changes that persist until adulthood, as higher body weight, accompanied by higher FFA (Bei et al., 2015) and total cholesterol, as well as fat depots (Hahn, 1984; Enes-Marques et al., 2020), without changes in plasma TG (Rodrigues et al., 2007; Conceição et al., 2013).

Interestingly, besides dyslipidemia, adult animals with childhood obesity also presented lipid accumulation in the liver, observed by increased liver TG content, accompanied by increased DNL, seen by enhanced expression of FASN in the liver, following previous studies (Duff and Snell, 1982; Ribas-Aulinas et al., 2021; Yang et a., 2018). Indeed, increased content of liver TG, as well as plasma levels of TG and FFA, might be ascribed to increased expression of FASN in the liver in SL animals, which could account for increased synthesis of fatty acids and consequently DNL.

In addition to higher body weight and impairment in lipidic profile, the SL animals also had a higher concentration of circulating glucocorticoids, as previously shown (Hou et al., 2011; de Souza et al., 2022). Animals with obesity in different experimental models have increased levels of circulating glucocorticoids (Freedman et al., 1986; Mantha et al., 1999; Tokuyama and Himms-Hagen, 1989), and removal of them by adrenalectomy attenuates or prevents the development of obesity (Romsos, 1991; Tokuyama and Himms-Hagen, 1989; Freedman et al., 1986), pointing glucocorticoids as a possible mediator of obesity in these experimental models. Accordingly, the role of glucocorticoids as an important modulator of energy balance in obesity induced by early overnutrition was also demonstrated in the current work, where ADX was able to reduce body weight gain and food intake, to induce an improvement in lipid profile, with reduction of FFA, TG, total and LDL cholesterol, and to reduce TG content as well as expression of FASN in the liver of SL animals, and treatment with corticosterone could restore most of these changes induced by ADX.

ADX-induced reduction of body weight and plasma lipid profile in childhood obesity is following the reversal effect of ADX on metabolic parameters in other obesity models (Alarrayed et al., 1992; Mantha et al., 1999; Kibenge and Chan, 2001; Yilmaz et al., 2002). Additionally, the decrease of triglyceride content and FASN expression in the liver by ADX may be due to the well-established stimulatory effects of glucocorticoids on both the accumulation of lipids and TG in the liver (Suzuki and Mizugashira, 1979) and the expression of key enzymes of DNL in the liver, as FASN (Wang et al., 2004; Lee et al., 2011). It is tempting to suggest that enhanced levels of circulating glucocorticoids in SL animals might have permissive effects on FASN expression, and consequently DNL, and TG accumulation in the liver, since ADX was able to attenuate these parameters in the present work, and glucocorticoids are known to induce FASN and DNL (Wang et al., 2004; Lee et al., 2011), and TG content in the liver (Suzuki and Mizugashira, 1979). Conversely, *in vitro* studies showed that both lipid accumulation and TG content in HepG2 cells was significantly increased by glucocorticoid treatment by an increase of DNL (Yang et al., 2018). Thus, the lack of effects of litter size reduction and adrenal removal on the other molecules involved in lipid metabolism in the liver is likely to indicate that FASN is a key molecule underlying glucocorticoids-induced upregulation of DNL and TG accumulation in the liver on adult male rats with childhood obesity.

Despite plasma insulin was not measured, it is known that animals reared in small litters present increased concentrations of circulating insulin (Hou et al., 2011; Conceição et al., 2013; Liu et al., 2013), and insulin acts in lipid metabolism, stimulating DNL mainly by upregulating the transcription factor sterol regulatory element binding protein (SREBP)-1c for expression of ACC and FASN (Horton et al., 2002; Csaki and Reue, 2010), important for fatty acid synthesis and lipid biosynthesis. In addition to this, insulin regulates glucose metabolism in the hepatocytes, stimulating glycolysis and synthesis of glycogen and inhibiting gluconeogenesis (Titchenell et al., 2017). For these outcomes, insulin binds to its receptor, insulin receptor (IR) in the membrane, a tyrosine kinase receptor which autophosphorylates itself in the internal surface of the cell upon insulin binding, and thereby activates its intrinsic tyrosine kinase activity, resulting in phosphorylation of substrates of insulin receptor (IRS) proteins on tyrosine residues, which can activate phosphatidylinositol- 3- kinase (PI3K). PI3K is a dimer composed of a catalytic (p110) and a regulatory (p85) subunit, and the target proteins of PI3K are Akt and PKC isoforms (Backer et al., 1992; Czech and Corvera, 1999; Lietzke et al., 2000). In this context, the reduction of the expression of PI3K in the liver of adult animals with neonatal overnutrition observed in the present work is according to impairment of hepatic PI3K pathway in neonatal overfed animals in adulthood shown in previous studies (Conceição et al., 2013; Huang et al., 2020), despite the other molecules of this pathway was unchanged in the current data, suggesting a partial disruption of the insulin signaling pathway in the liver of adult male rats with neonatal overnutrition.

GLUT2 in the liver is responsible for the influx of glucose in the postprandial period, and the efflux of glucose in the post-absorptive and fasting periods (Chadt and Al-Hasani, 2020). In addition to the insulin signaling pathway, the lack of changes in the expression of GLUT2 in the liver by either litter size reduction or adrenalectomy also points that increased DNL observed in SL animals is not related to insulin-induced glucose utilization in the liver, since PI3K-AKT pathway in this tissue was impaired in animals with childhood obesity. On the other hand, a decrease of IRS2 expression in the liver following ADX might underly reduction of FASN expression and TG content in this tissue, since IRS2 in the liver has been pointed as an important mediator of lipid synthesis and DNL in the liver (Eckstein et al., 2017).

The major function of the 11β-HSD1 is to convert inactive cortisone to active cortisol (corticosterone). This is in the endoplasmic reticulum and widely expressed in liver and adipose tissue, being responsible for amplifying local glucocorticoids action (Jamieson et al., 1995; Bujalska et al., 1997; Masuzaki et al., 2001). The majority of obesity animal models show that hepatic 11β- HSD1 expression and/or activity are unchanged (Morton et al., 2004) or reduced (Drake et al., 2005). Despite Yang et al. (2018) have reported that postnatal overfeeding-induced litter size reduction leads to increased mRNA expression of 11β-HSD1 in the liver of adult male rats, the present data indicate the opposite, hepatic protein expression of 11β-HSD1 being downregulated by neonatal overnutrition. Accordingly, it is likely that hepatic lipid accumulation during adulthood in animals with lactation overnutrition may be ascribed to increased circulating glucocorticoids, rather than glucocorticoids overexposure through 11β-HSD1 upregulation in the liver. This reduction in liver 11β-HSD1 may account for partial impairment of the insulin signaling pathway in the liver in the present study, once overexpression of 11β -HSD1 in the liver in mice results in insulin resistance (Masuzaki et al., 2001; Paterson et al., 2004).

In spite of recovery of body weight in the SL/ADX + B group without recovery of food intake was unexpected, it seems to be a specific profile of SL animals to some challenges, since Marangon et al. (2020) have reported that SL animals also showed changes on body weight gain without changes on food intake. Within this, context, the lack of corticosteronemia elevation in the SL/ADX + B group, in spite of the highest values of fluid intake, is likely because SL animals might have been adapted to increased corticosterone plasma levels, and that the concentration of corticosterone treatment of 25 mg/L, which was enough to restore corticosterone plasma levels to NL animals, could not be sufficient to restore corticosterone levels in SL animals. Accordingly, the treatment with corticosterone was, at least in part, effective in reversing or attenuating the effects of ADX even in SL animals, since some parameters have been partially or fully restored in ADX + B animals. However, it cannot be ruled out the possibility that other hormones of adrenal glands, as aldosterone, could also participate on these responses. In fact, replacement with aldosterone + cortiscosterone to ADX rats was more effective in enhancing food intake and body weight gain than only corticosterone replacement (Devenport et al., 1983; 1985). Furthermore, aldosterone replacement to ADX animals restored daily caloric intake, especially lipid intake, and body fat deposition (Devenport and Devenport, 1982; Tempel and Leibowitz, 1989). Altogether, it is important to point out that, besides corticosterone, aldosterone is another adrenal hormone important to be considered in the control of energy balance. Finally, it is important to highlight that a limitation of the current study was that female animals were not included in the analysis; thus, the present conclusion is only to male rats, since male and female animals may have different responses.

## Conclusion

In summary, metabolic and liver dysfunctions in adult male rats induced by neonatal overnutrition were attenuated by ADX, and the treatment with corticosterone restored most of them. Altogether, these data show that, likely in other obesity models, in childhood obesity, glucocorticoids also have permissive effects to induce hyperlipidemia and TG accumulation in the liver, probably by specific upregulation of one of the key enzymes of DNL: FASN. In conclusion, the role of glucocorticoids in the metabolic impairments in obesity should be considered, and possibly be a target for interventions in obesity and metabolic syndrome.

## Data Availability

The raw data supporting the conclusion of this article will be made available by the authors, without undue reservation.
