# Supplementary material for: Glucocorticoids contribute to metabolic and liver impairments induced by lactation overnutrition in male adult rats
Source: Front Physiol. 2023 May 10;14:1161582. doi: 10.3389/fphys.2023.1161582 (PMC10206267; doi:10.3389/fphys.2023.1161582)
Supplement: Supplementary file 1 [file DataSheet2.PDF]

Primary antibodies used in Western blotting.

**Target protein**	**Catalog #**	**Dilution**
Vinculin	sc-73614	1:500
Phospho-Insulin receptor subunit beta (p-Irβ)	GTX25681	1:1000
Insulin receptor subunit beta (Irβ)	#30255	1:1000
Insulin receptor substrate 1 (IRS1)	sc-559	1:1000
Insulin receptor substrate 2 (IRS2)	sc-1555	1:500
Phosphatidylinositol 3-kinasep110α ($_{p110\alpha}$PI3K)	#4255	1:1000
Protein kinase B (AKT)	sc-8312	1:1000
Phosphorilated Protein kinase B (^{Ser473}p-AKT)	sc-7985	1:1000
Glucose transporter 2 (GLUT2)	sc-9117	1:500
Acetyl-CoA carboxylase (ACC)	#3676	1:1000
Phospho-Acetyl-CoA Carboxylase (Ser79) (Ser79pACC)	#11818	1:1000
Fatty acid synthase (FASN)	ab22759	1:1000
Diglyceride acyltransferase 2 (DGAT2)	sc-66859	1:1000
Apolipoprotein B100 (APOB100)	MABS2046	1:1000
11β-hydroxysteroid dehydrogenase type 1 (11β-HSD1)	sc-20175	1:1000
