# Supplementary material for: Glucocorticoids contribute to metabolic and liver impairments induced by lactation overnutrition in male adult rats
Source: Front Physiol. 2023 May 10;14:1161582. doi: 10.3389/fphys.2023.1161582 (PMC10206267; doi:10.3389/fphys.2023.1161582)
Supplement: Supplementary file 2 [file DataSheet1.PDF]

Gel Maps

NL (lanes 1–3) | SL (lanes 4–6) | NL (lanes 7–9) | SL (lanes 10–12)

Ladder – 1 – 2 - 3 - 4 - 5 - 6 - 7 - 8 - 9 - 10 - 11 - 12 - 13 - 14

1) Ladder – sham – ADX – ADX+B – sham – ADX – ADX+B – sham – ADX – ADX+B – sham – ADX – ADX+B - sham NL – ADX NL
2) Ladder – sham – ADX – ADX+B – sham – ADX – ADX+B – sham – ADX – ADX+B – sham – ADX – ADX+B - sham SL – ADX SL
3) Ladder – sham – ADX – ADX+B – sham – ADX – ADX+B – sham – ADX – ADX+B – sham – ADX – ADX+B – ADX+B NL – ADX+B SL
4) Ladder – sham – ADX – ADX+B – sham – ADX – ADX+B – sham – ADX – ADX+B – sham – ADX – ADX+B – ADX+B NL – ADX+B SL

Original blots

ApoB100; IRS1; p-IR_{β}, IR_{β}; ^{Ser473}p-AKT; AKT; DGAT2; Vinculin

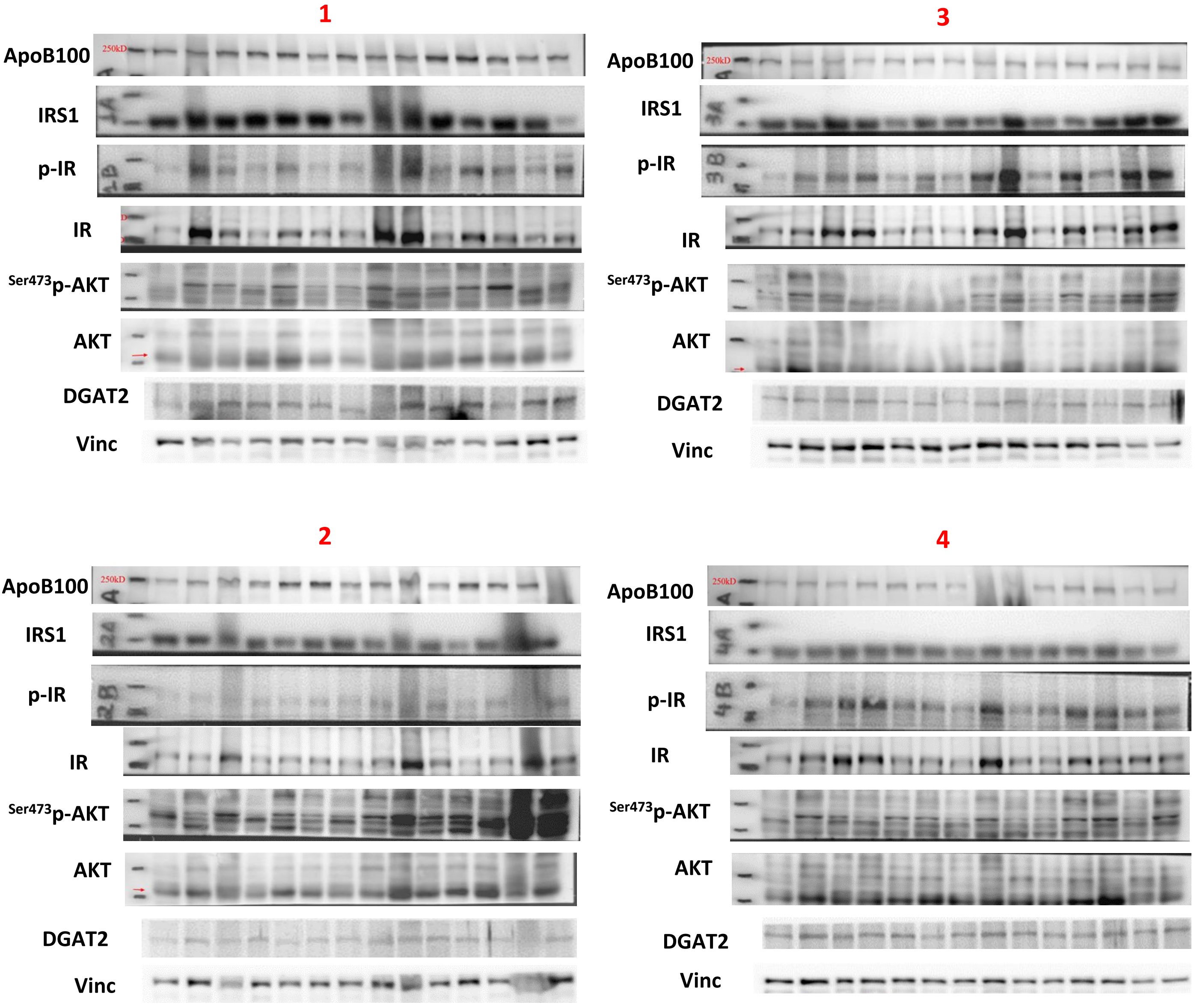

Representative blots:
ApoB100: gel#1, samples 7 – 12
IRS1: : gel#3, samples 1 – 6
pIR: gel#3, samples 1 - 6
IR: gel#3, samples 1 – 6
p-AKT: gel#1, samples 1 – 6
AKT: gel#1, samples 1 – 6
DGAT2: gel#4, samples 1 - 6
*Vinculin to each correspondent samples described above.

Gel Maps

	NL			SL			NL			SL				
Ladder –	1 –	2 -	3 -	4 -	5 -	6 -	7 -	8 -	9 -	10 -	11 -	12 -	13 -	14

1) Ladder – sham – ADX – ADX+B – sham – ADX – ADX+B – sham – ADX – ADX+B – sham – ADX – ADX+B - sham NL – ADX NL
2) Ladder – sham – ADX – ADX+B – sham – ADX – ADX+B – sham – ADX – ADX+B – sham – ADX – ADX+B - sham SL – ADX SL
3) Ladder – sham – ADX – ADX+B – sham – ADX – ADX+B – sham – ADX – ADX+B – sham – ADX – ADX+B – ADX+B NL – ADX+B SL
4) Ladder – sham – ADX – ADX+B – sham – ADX – ADX+B – sham – ADX – ADX+B – sham – ADX – ADX+B – ADX+B NL – ADX+B SL

Original blots

p-ACC; ACC; 11βHSD1; Vinculin

1
p-ACC
ACC
11βHSD1
Vinc

2
p-ACC
ACC
11βHSD1
Vinc

3
p-ACC
ACC
11βHSD1
Vinc

4
p-ACC
ACC
11βHSD1
Vinc

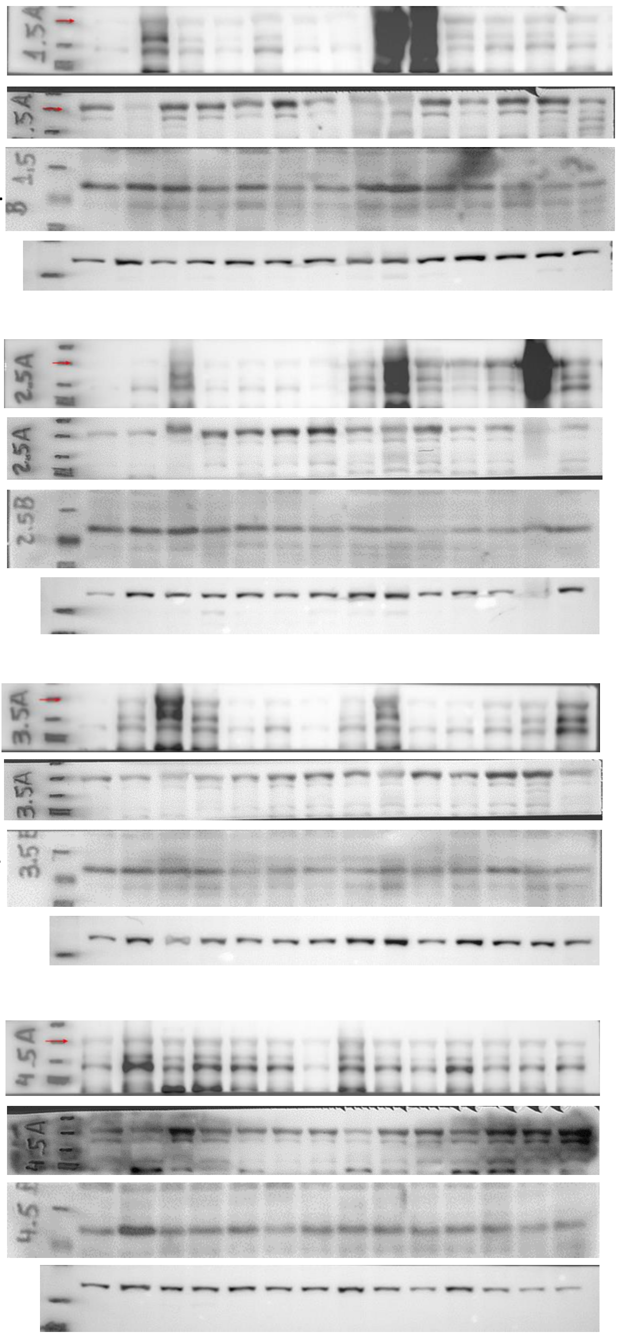

$_{p110\alpha}$PI3K; Vinculin

$_{p110}$PI3K
Vinc
$_{p110}$PI3K
Vinc
$_{p110}$PI3K
Vinc
$_{p110}$PI3K
Vinc

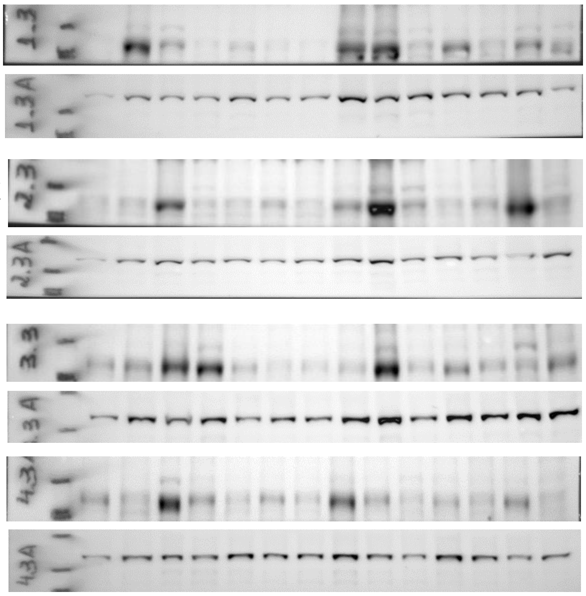

GLUT2; Vinculin

GLUT2
Vinc
GLUT2
Vinc
GLUT2
Vinc
GLUT2
Vinc

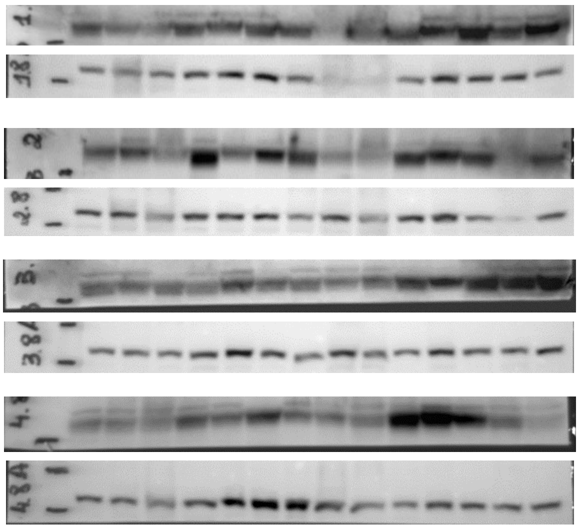

Representative blots:
p-ACC: gel#4, samples 7 - 12
ACC: gel#4, samples 7 – 12
11BHSD1: gel#2, samples 1 - 6
$_{p110}$PI3K: gel#3, samples 7 - 12
GLUT2: gel#3, samples 1 - 6
*Vinculin to each correspondent samples described above.

Gel Maps

NL (1–3) | SL (4–6) | NL (7–9) | SL (10–12)

Ladder – 1 – 2 - 3 - 4 - 5 - 6 - 7 - 8 - 9 - 10 - 11 - 12 - 13 - 14

1) Ladder – sham – ADX – ADX+B – sham – ADX – ADX+B – sham – ADX – ADX+B – sham – ADX – ADX+B - sham NL – ADX NL
2) Ladder – sham – ADX – ADX+B – sham – ADX – ADX+B – sham – ADX – ADX+B – sham – ADX – ADX+B - sham SL – ADX SL
3) Ladder – sham – ADX – ADX+B – sham – ADX – ADX+B – sham – ADX – ADX+B – sham – ADX – ADX+B – ADX+B NL – ADX+B SL
4) Ladder – sham – ADX – ADX+B – sham – ADX – ADX+B – sham – ADX – ADX+B – sham – ADX – ADX+B – ADX+B NL – ADX+B SL

Original blots

FASN; Vinculin

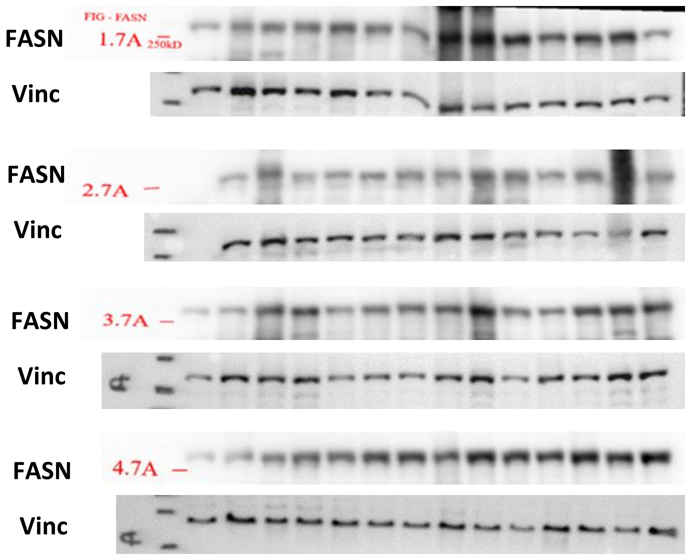

IRS2; Vinculin

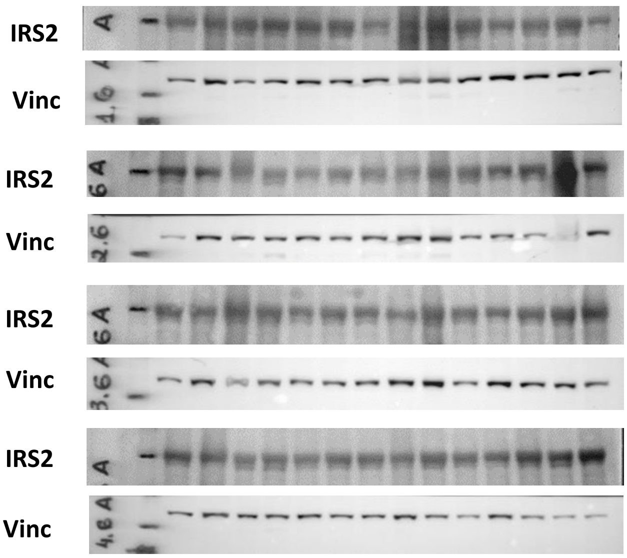

Representative blots:
FASN: gel#4, samples 7 - 12
IRS2: gel#4, samples 7 – 12
*Vinculin to each correspondent samples described above.
